# Establishing a Dose-Response Relationship Between Opioid Use and Hypogonadism: A Retrospective Case-Control Study

**DOI:** 10.31486/toj.20.0103

**Published:** 2021

**Authors:** Yashar Eshraghi, Natalie Hanks, Scott Rooney, Faeqeh Mir Yousefi Ata, Cruz Velasco, Maged Guirguis, Gabriel Uwaifo

**Affiliations:** ^1^ Department of Anesthesiology, Interventional Pain Management, Ochsner Clinic Foundation, New Orleans, LA; ^2^ The University of Queensland Faculty of Medicine, Ochsner Clinical School, New Orleans, LA; ^3^ Department of Academics, Faculty of Medicine, Eskisehir Osmangazi University, Eskisehir, Turkey; ^4^ Center for Applied Health Services Research, Ochsner Clinic Foundation, New Orleans, LA; ^5^ Department of Endocrinology, Diabetes, Metabolism and Weight Management, Ochsner Clinic Foundation, New Orleans, LA

**Keywords:** *Analgesics–opioid*, *hypogonadism*, *opioid-related disorders*

## Abstract

**Background:** Opioid-induced androgen deficiency (OPIAD) related to chronic, long-acting opioid use can be a significant detriment to patient quality of life. The aim of this study was to investigate the association between chronic opioid use and hypogonadism.

**Methods:** A single-center, retrospective, matched case-control analysis of 357 males (94 cases, 263 controls, aiming for 1:4 matching) was performed. Study subjects were ages 18 to 80 years and had a diagnosis of chronic opioid use. Patients with a hypogonadism diagnosis were matched to patients without a hypogonadism diagnosis by age, ethnicity, and body mass index. The maximum morphine equivalent daily dose (MEDD) was compared in each group.

**Results:** A significant linear association between MEDD and the odds of developing hypogonadism (*P*<0.001) in males with chronic use of opioids was observed, with an odds ratio of 1.44 (95% CI 1.16-1.78) by 100-unit difference in maximum MEDD.

**Conclusion:** Results showed a significant, positive linear association between chronic opioid dose and the odds of developing hypogonadism in males. This higher index of suspicion of OPIAD could lead to earlier recognition of symptoms and increase the positive predictive value of diagnostic laboratory tests.

## INTRODUCTION

One of the less commonly appreciated, detected, and recognized complications of opioid use is hypogonadotropic hypogonadism. Opioid-induced androgen deficiency (OPIAD) was first documented in 1973, when males addicted to heroin or methadone were noted to have lower levels of testosterone.^1^ More recent studies have continued to demonstrate a high rate of both hypotestosteronemia and clinically significant hypogonadism in males taking opioids for chronic pain or maintenance therapy for addiction.^2-5^ Although many opioid adverse effects are well known and readily identified in clinic, the effect opioids have on the endocrine system are commonly overlooked and underappreciated, even though their prevalence is likely fairly high with potential quality of life and health-related consequences.^6-9^

The negative side effects of chronic opioid use on the endocrine system are significant in the adult population but not adequately characterized in the medical literature. Androgen deficiency, which is one of the more dominant endocrine consequences of chronic opioid use, has serious morbidity potential, including fatigue, muscle atrophy (sarcopenia), decreased libido, erectile dysfunction, depressed mood with anhedonia, reduced quality of life, anemia, subfertility or infertility, gynecomastia, and decreased bone density, which can lead to osteoporosis and bone fractures.^10,11^ These complications can cause substantive distress for patients and are major expenses for health care systems.^12,13^ As narcotic abuse continues to rise, elucidating the consequences of continuous opioid use becomes crucial. Studies to date lack the statistical power to adequately investigate a dose-response relationship between opioids and hypogonadism. Examining this relationship is necessary to help identify which patients on opioids need to be screened for these complications and to guide adequate and safe treatment of such patients. The dose-response relationship between opioids and hypogonadism could also reshape clinical guidelines for the diagnosis, management, and/or treatment of OPIAD.

With this retrospective medical record review, we aimed to characterize the prevalence of male hypogonadism among chronic opioid users and the dose-effect relationship between opioids and hypogonadism.

## METHODS

### Study Design

This retrospective case-control study using health data from the Ochsner Health Epic electronic health record (EHR) database was approved by the Institutional Review Board of Ochsner Health with waiver of consent (#STUDY00000714). Patients’ medical records were reviewed to collect data on demographics, medications, and inpatient and outpatient diagnoses.

### Study Population

**Figure 1.**
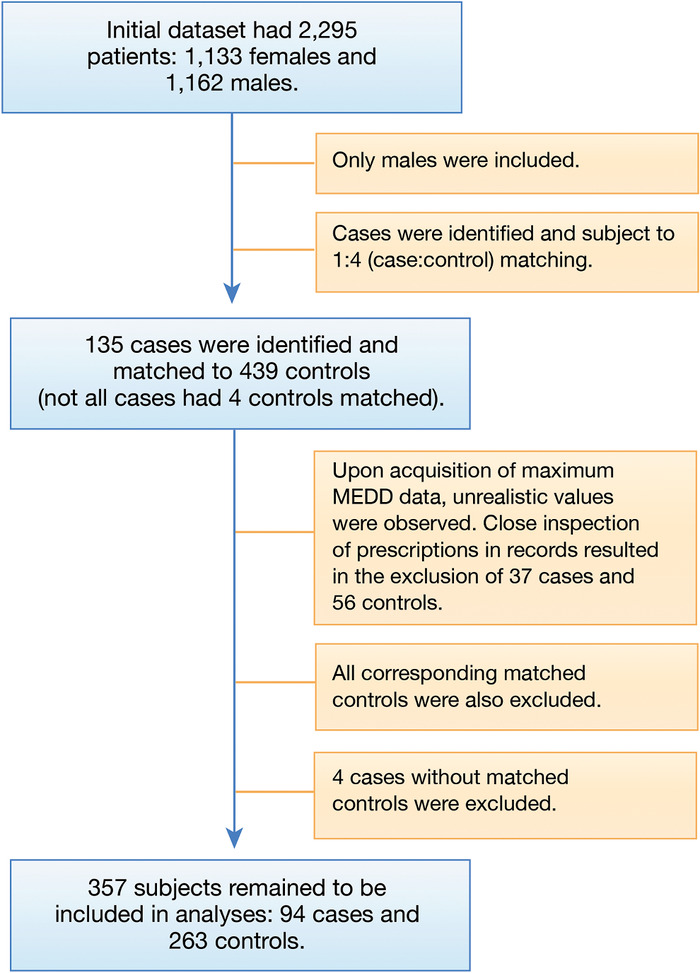
**Data flow diagram demonstrates subject selection for the study.** MEDD, morphine equivalent daily dose.

We identified 1,162 males aged 18 to 80 years who had ≥90 days of opioid use from January 2012 to May 2019 (Figure 1). The cutpoint of ≥90 days was selected because it is a well-established clinical threshold of prolonged opioid use. We excluded males who had a diagnosis of Klinefelter syndrome, other chromosomal abnormalities, cryptorchidism, varicocele, myotonic dystrophy, prior mumps infection, radiation to the testes, testicular torsion, long-term corticosteroid use, prostate cancer, or other known relevant endocrine disorder. These exclusions narrowed the cohort to 574 subjects. Of the 574 subjects, 135 carried a chart diagnosis of hypogonadism or low testosterone. Patients were excluded from the study if they were diagnosed with hypogonadism or low testosterone before their chronic opioid use.

For the control group, we identified 439 subjects who never had a diagnosis of hypogonadism or low testosterone and followed the aforementioned exclusion criteria. Cases were matched (aiming for 1:4 matching) to controls by age, ethnicity, and body mass index (BMI). We used the greedy method, which matches one or more controls for each case; controls may be matched to cases by one or more covariates. Once a match is made using this method, it cannot be broken. The greedy method was implemented by using the SAS macro gmatch^14^ (matching weights=3, 2, 1 and largest difference compatible with a valid match=0, 1, 3 for race, age, and BMI, respectively, and with weighted sum distance). Four cases did not have matching controls, so they were excluded from the study.

### Measurements

The Ochsner Epic EHR automatically calculates the morphine equivalent daily dose (MEDD) for each opioid prescription. After examination of the MEDD data for the cohort, we found several MEDDs to be >10,000. Each >10,000 value was associated with a prescription for either methadone or buprenorphine. Because methadone increases in potency with opioid-tolerant patients and buprenorphine acts as a partial opioid agonist, calculating the MEDD varies. Because of these additional complexities, patients taking methadone or buprenorphine were excluded from the study, resulting in the exclusion of 37 cases and 56 controls. After matching, a total cohort of 357 subjects was used in the study: 94 cases and 263 controls.

### Statistical Analysis

The observed prevalence of hypogonadism by groups of matching variables (before matching) and for ranges of MEDD postmatch were calculated. The association between hypogonadism and MEDD was evaluated by conditional logistic regression, with linear terms of continuous MEDD. To account for confounding variables, we matched cases to controls by age, ethnicity, and BMI. A nonlinear association of MEDD with hypogonadism was illustrated with restricted cubic splines. Analyses were performed using SAS/STAT statistical software, version 14.2 (SAS Institute, Inc). This manuscript adheres to the application of the Strengthening the Reporting of Observational Studies in Epidemiology (STROBE) guidelines.

## RESULTS

Table 1 displays the prevalence of hypogonadism among the total 1,162 male subjects with chronic opioid use by age, race, and BMI groups. Among subjects in the age groups of 18 to 39 years, 40 to 65 years, and >65 years, 7.2%, 13.7%, and 13.1%, respectively, were diagnosed with hypogonadism. Among subjects diagnosed with hypogonadism, 3.8% were underweight (BMI <18.5 kg/m^2^), 11.4% were normal weight (BMI 18.5 to 24.9 kg/m^2^), 10.9% were overweight (BMI of 25 to 29.9 kg/m^2^), and 15.1% were obese (BMI >30 kg/m^2^).

**Table 1.** Prevalence of Hypogonadism in the Study Cohorts by Age, Race, and Body Mass Index

Variable	All Patients, n=1,162^a^	Patients Without Hypogonadism, n=1,016^b^	Patients With Hypogonadism, n=146^b^
Age, years			
18-39	167 (14.4)	155 (92.8)	12 (7.2)
40-65	621 (53.4)	536 (86.3)	85 (13.7)
>65	374 (32.2)	325 (86.9)	49 (13.1)
Race			
White	828 (71.3)	723 (87.3)	105 (12.7)
Black	269 (23.1)	231 (85.9)	38 (14.1)
Other	65 (5.6)	62 (95.4)	3 (4.6)
Body mass index, kg/m^2^			
Underweight, <18.5	26 (2.2)	25 (96.2)	1 (3.8)
Normal, 18.5-24.9	263 (22.6)	233 (88.6)	30 (11.4)
Overweight, 25-29.9	396 (34.1)	353 (89.1)	43 (10.9)
Obese, ≥30	477 (41.0)	405 (84.9)	72 (15.1)
Obese Class 1, 30-<35	271 (23.3)	234 (86.3)	37 (13.7)
Obese Class 2, 35-<40	122 (10.5)	102 (83.6)	20 (16.4)
Obese Class 3, ≥40	84 (7.2)	69 (82.1)	15 (17.9)

^a^Percentages are calculated by column.

^b^Percentages are calculated across the row.

Note: Data are presented as n (%).

From the conditional logistic regression with continuous MEDD as a predictor, we observed a linear association of developing hypogonadism (*P*<0.001) with an odds ratio of 1.44 (95% CI 1.16-1.78) for each 100-unit difference in MEDD. Thus, we observed 44% (95% CI 16%-78%) increased odds of developing hypogonadism between patients having a 100-unit difference in MEDD.

**Figure 2.**
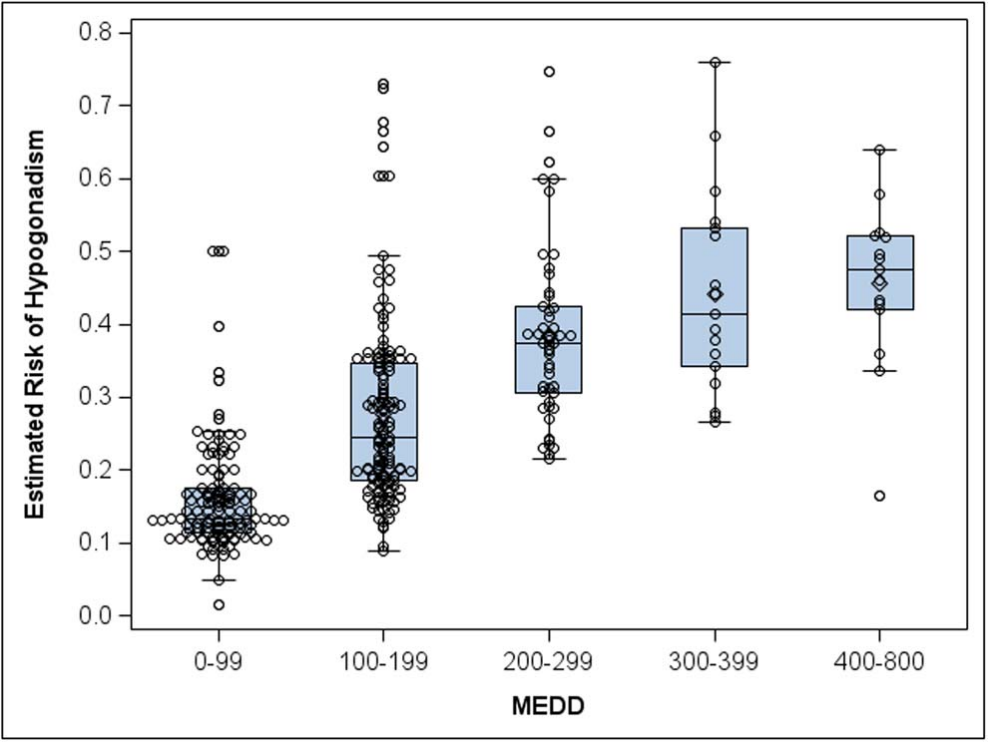
Estimated risk of hypogonadism for patients at various ranges of morphine equivalent daily dose (MEDD). The circles are an estimated risk of hypogonadism for each subject from a conditional logistic regression model on MEDD. The figure displays a rise followed by a plateau in average risk of hypogonadism with increasing MEDD.

**Figure 3.**
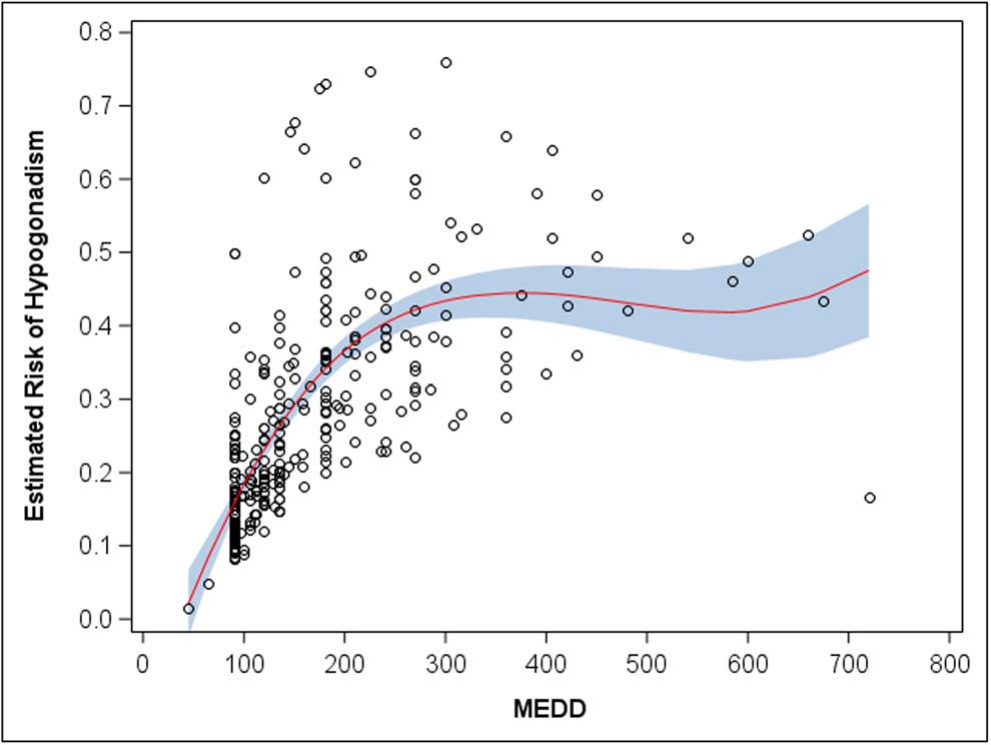
**Estimated risk of hypogonadism as a function of continuous morphine equivalent daily dose (MEDD). The circles are an estimated risk of hypogonadism for each subject from a conditional logistic regression model on MEDD. The red line is the average estimated risk of hypogonadism as a function of MEDD. The blue band represents the confidence intervals of estimated risk. Wider confidence intervals reflect smaller sample sizes and thus less certainty about the average estimated risk of hypogonadism.** (A color version of this figure is available at https://doi.org/10.31486/toj.20.0103.)

Table 2 shows the observed prevalence of hypogonadism for intervals of MEDD. The plateau from 400 to 800 MEDD is based on a limited sample size (n=14). From a logistic regression model with restricted cubic splines on MEDD, we found a nonlinear association with the odds of hypogonadism status (*P*=0.015). Figure 2 depicts the nonlinear association as an increase and plateau of the estimated risk of hypogonadism for intervals of MEDD. Figure 3 demonstrates the same estimated risk of hypogonadism as a smoothed function of continuous MEDD. Both Figures 2 and 3 show an increased estimated risk of hypogonadism as the MEDD increases.

**Table 2.** Prevalence of Hypogonadism for Each Morphine Equivalent Daily Dose (MEDD) Range

MEDD Range	Total Patients, n=356	Study Group With Hypogonadism, n=93^a^	Control Group Without Hypogonadism, n=263
0-99	135	21 (15.6)	114 (84.4)
100-199	140	38 (27.1)	102 (72.9)
200-299	53	22 (41.5)	31 (58.5)
300-399	14	5 (35.7)	9 (64.3)
400-800	14	7 (50.0)	7 (50.0)

^a^One patient had a MEDD >800 and is not included in the analysis.

Note: Percentages are calculated by row. Data are presented as n (%).

## DISCUSSION

Our primary results show that chronic opioid users were more likely to have hypogonadism with higher daily doses of prescribed opioids. This positive trend was highly statistically significant. Our findings are consistent with prior studies demonstrating an increased prevalence of androgen deficiency among chronic opioid users, but to our knowledge, this study is the first to demonstrate a significant dose-effect relationship between opioids and the development of hypogonadism.^10^ As is apparent from our findings and previous studies, at least a fraction of this observed association between hypogonadism and opioid use is causal and therefore amenable to intervention.

The side effects of hypogonadism related to chronic opioid use can be a significant burden to patient quality of life and the health care system. However, the negative sequelae of chronic opioid use on the endocrine system are often poorly investigated, frequently unrecognized, and commonly left untreated.^15^ As of 2021, no guidelines are available for the recognition and diagnosis of OPIAD. Studies have demonstrated an increased prevalence of OPIAD in patients receiving opioid therapy for longer than a few weeks, as well as an increased prevalence in patients receiving a median dose >100 MEDD.^16,17^ Evaluation of any patient manifesting symptoms of hypogonadism for signs of OPIAD, such as reduced libido, erectile dysfunction, depression, fatigue, or night sweats, is appropriate. Laboratory testing to assess for the presence of opioid-induced hypogonadism, including total testosterone, free testosterone, sex hormone binding globulin, luteinizing hormone, follicle-stimulating hormone, dehydroepiandrosterone sulfate, and estradiol, may be warranted if clinical suspicion is high enough. Treatment options include lifestyle changes in diet and exercise, tapering of opioids, opioid rotation, or hormonal replacement therapies if symptoms are significant enough.^3,18,19^

Although OPIAD is studied primarily in the male population, future studies should expand the patient population examined to include females. Opioid-induced hypogonadism affects the female population in similar ways as males. Females have comparable symptoms, as well as hot flashes and irregular menstruation. Examining if a dose-response relationship between chronic long-acting opioid use and hypogonadism exists in females could be important. Because this study demonstrates a positive correlation between the daily dose of prescribed opioids and the prevalence of hypogonadism, clinicians should have an increased index of suspicion for the development of hypogonadism in patients using opioids chronically. This higher index of suspicion could lead to earlier recognition of symptoms, increase the positive predictive value of diagnostic laboratory tests, and potentially assist in the formation of clinical guidelines for the diagnosis and management of OPIAD.

Important limitations of this study include its retrospective nature and the inability to document causality between opioid exposure and development of OPIAD. The retrospective nature of our study also limits our capacity to rigorously distinguish between low testosterone as a biochemical finding vs clinical hypogonadism as a clinical syndrome. MEDD data were taken from the EHR and may not represent the dosages patients were taking. The degree to which OPIAD can impact both short-term and long-term fertility, especially in younger patients using opioids, is another important clinical question that our study was not designed to answer. Finally, although our study includes one of the largest reported cohorts in this area of investigation, the sample size is still limited relative to the patient population exposed to opioids. Our study was not able to characterize the role and effects of methadone and buprenorphine use, as patients taking these drugs were excluded from our cohort, but these opioid receptor partial agonists are widely used in the chronic opioid abuse remediation population. These limitations highlight the urgent need for well-designed, possibly multicenter, prospective clinical studies to investigate these and other questions regarding OPIAD in both males and females on chronic opioid therapy.

## CONCLUSION

This single-center study demonstrates the linear dose-response relationship between chronic opioid use and hypogonadism. Recognition of this relationship leads to earlier recognition of symptoms, increases the positive predictive value of diagnostic laboratory tests, and potentially assists in developing formal clinical guidelines for the diagnosis and management of OPIAD in future studies.

## Notes

*This article meets the Accreditation Council for Graduate Medical Education and the American Board of Medical Specialties Maintenance of Certification competencies for Patient Care, Medical Knowledge, and Practice-Based Learning and Improvement.*
